# Assessing the nutritional needs of men with prostate cancer

**DOI:** 10.1186/s12937-019-0506-7

**Published:** 2019-12-02

**Authors:** Kaitlin McLaughlin, Lindsay Hedden, Philip Pollock, Celestia Higano, Rachel A. Murphy

**Affiliations:** 1 School of Population and Public Health, University of British Columbia, Vancouver, BC Canada; 2 Centre for Clinical Epidemiology and Evaluation, University of British Columbia, Vancouver, BC Canada; 3 Vancouver Prostate Centre, Vancouver, BC Canada; 4 Department of Urologic Sciences, University of British Columbia, Vancouver, BC Canada; 5 University of Washington, Fred Hutchinson Cancer Research, Seattle, WA USA

**Keywords:** Nutrition services, Supportive care, Survivorship, Cancer, Prostate cancer, Diet and cancer

## Abstract

**Background:**

Nutrition is important for prostate cancer (PC) survivorship care to help achieve a healthy weight, reduce treatment side effects and reduce the risk of developing other chronic diseases. We aimed to advance the understanding of the nutritional needs of men with PC and services that could be potentially implemented to address them.

**Methods:**

We conducted a needs assessment of nutrition services for men with PC drawing on four perspectives; 1) patient evaluation of a nutrition education session in British Columbia (BC), 2) survey of BC health professionals, 3) an environmental scan of existing nutrition services across Canada and 4) a scoping literature review.

**Results:**

Patients expressed a need for more nutrition information and a desire for additional nutrition services. More than 60% of health professionals believed there is a need for more nutrition services for men with PC, and reported the focus should be on weight management or management of PC progression. The environmental scan revealed few existing services for men with PC across Canada, most were inclusive of multiple cancers and not tailored for men with PC. Eighteen completed studies were identified in the scoping literature review. The majority provided combined diet and exercise programs with various formats of delivery such as individual, group and home-based. Overall, 78% of studies reported improvements in one or more of the following measures: dietary intake/ diet quality, body composition, self-efficacy, quality of life, fatigue, practicing health behavior goals and physical function/ exercise. Four studies assessed feasibility, adherence or satisfaction with all reporting positive findings.

**Conclusion:**

Despite the high prevalence of PC in Canada, and the perceived need for more support by patients and health professionals, there are limited nutrition services for men with PC. Evidence from the literature suggests nutrition services are effective and well-accepted by men with PC. Our findings define a need for standardized nutrition services for men with PC that assess and meet long term nutritional needs. Our findings also provide insight into the type and delivery of nutrition services that may help close the gap in care for men with PC.

## Background

In 2018, an estimated 1.3 million men were diagnosed with prostate cancer (PC) worldwide [1]. PC is the second most common cancer in men, and is particularly prevalent among developed countries [1]. Over the past decade there have been substantial improvements in cancer care including earlier detection, management and treatment leading to declining PC mortality and a 5-year relative survival rate of 95% [2]. Supportive care for men with PC and their families is important since many men may face physical, psychological and psychosocial challenges following a PC diagnosis and therapy for sexual and urinary dysfunction, fear of cancer reoccurrence and development of other chronic diseases [3]. Due to enhanced PC care, men are more likely to die from cardiovascular or respiratory diseases, rather than from PC itself [4–7].

Diet also plays an important role in the prevention of chronic diseases (e.g. diabetes, cardiovascular disease and osteoporosis) that men with PC can be at increased risk of developing, particularly with the use of androgen deprivation therapy (ADT) which is associated with increased fat mass and insulin resistance [8–10]. For instance, a study of over 73,000 men with prostate cancer found those treated with ADT had a 44, 16, 11 and 16% increased risk of developing diabetes, coronary heart disease, myocardial infarction and sudden cardiac death, respectively [9]. High levels of obesity are prevalent among cancer survivors, often at similar levels as the general population [11]. Most studies also report less healthy diets among male cancer survivors, including lower fruit and vegetable intake compared to breast and uterine cancer survivors [12, 13]. As a result, the American Cancer Society’s Prostate Cancer Survivorship Care Guidelines emphasize the need for nutrition as a part of survivorship care for men with PC [3]. Furthermore, men diagnosed with PC may be motivated to make dietary changes after a cancer diagnosis [14]. A large body of evidence shows that lifestyle change, including dietary modifications are feasible for men with PC [15], may minimize side effects of PC treatment [4], help achieve a healthy body mass index [16–19], and improve health related quality of life [20]. Studies report that men with PC value access to dietary advice and use dietary change as a way to regain control over their diagnosis and to enhance survivorship [21, 22]. Together, this evidence highlights the importance of delivering nutrition services for men with PC to provide the knowledge and support needed to make lifestyle changes to improve quality of life and minimize the effects of PC and related treatment.

Despite the need for nutrition in supportive PC care, there are barriers to delivering nutrition services [23]. This may be particularly true in a public health care system. In Canada, many hospitals prioritize access to nutrition services due to limited funding and thus access to dietitians. For example, at many provincial cancer agencies, patients are referred to a dietitian only if they meet criteria for malnutrition which is generally based on a history of recent weight loss or if patients experience severe side effects of cancer treatment that impact dietary intake, both of which are uncommon among men with PC [24]. Several Canadian provinces operate free telephone-based services supported by provincial government that connect people with dietitians [25–28]. However, there are a limited number of dietitians with expertise in oncology and they provide services for all types of cancers (e.g. there is one oncology dietitian at HealthLink BC that serves the 4.6 million people in British Columbia (BC)).

As a result, within a public healthcare system, access to nutritional services is often limited for men with PC. Indeed, there is no standard nutrition education program for PC across BC. This, along with the evidence from the published literature, suggests there is a missed opportunity to support the needs of men with PC, and encourage lifestyle change to improve PC outcomes and overall health. New healthcare programs that draw on additional sources of funding, may be a means to provide more comprehensive support to patients. The Prostate Cancer Supportive Care (PCSC) Program [29], that includes education on lifestyle changes for diet and exercise, was established in 2013 and is now being expanded to cancer centres across BC in partnership with the provincial cancer agency (BC Cancer). Currently, diet and nutrition information are provided in the PCSC Program through a single group education session. However, it is unlikely that this education session alone adequately meets the needs of men with PC. An understanding of the nutritional needs of men with PC, nutrition services available to men with PC in other healthcare settings and services that could be implemented from the evidence base is critical for informing supportive care delivery within the PCSC Program and supportive care programs more broadly. We conducted a needs assessment of nutritional services for men with PC that drew on four areas; 1) patient evaluation of the current PCSC nutrition education session, 2) a health professional survey regarding nutritional services, 3) an environmental scan of existing nutritional services for men with PC in Canada, and 4) a scoping literature review of nutrition services for men with PC.

## Methods

### Evaluation of the PCSC Program’s “Nutrition for Prostate Cancer Patients” education session

To provide patients’ perspectives on nutrition services, we analyzed existing patient feedback on the PCSC Program’s nutrition education session. All men diagnosed with PC and their families from the greater Vancouver area were eligible to attend the session after registering with the PCSC Program. The 2-h session was delivered at Vancouver General Hospital in BC by a Registered Dietitian from BC Cancer in a lecture-style format and included time for questions and answers. The topics included nutrition and exercise guidelines for cancer survivors [3], plant-based foods, dietary patterns, obtaining nutrients from foods not supplements, Eating Well with Canada’s Food Guide [30], and an overview of diet and dietary supplement intervention studies in PC [31]. Immediately after the session concludes, attendees were asked to complete an anonymous evaluation form consisting of five close-ended questions related to satisfaction and open-ended additional comments. As forms are anonymous, we did not collect demographic or clinical information from participants.

### BC Health professional survey

We developed a survey to seek perspectives on the perceived need for nutrition services from health professionals in BC (urologists, radiation oncologists, medical oncologists, and registered dietitians) caring for men with PC. BC-based researchers with expertise in PC-related nutrition research were also invited to take part. The survey consisted of six questions that asked about the importance of nutritional services for men with PC, the content of such services, and how and when these services should be delivered. The survey was developed for this research study and is presented in Additional file 1. BC Health Professional Survey Questions. The questions were pilot tested and refined for clarity before being distributed to the larger sample. Purposive sampling was used to identify 56 health professionals who were asked to complete the survey via email within a 12-week period (May 2017 to August 2017). Reminder emails were sent to encourage participation.

### Environmental scan of existing nutritional services for men with PC

To understand existing nutrition services available for men with PC in Canada, we conducted an environmental scan of services. Appropriate resources were those that provided information on services of interest defined a priori as those directly related to nutrition or diet that were provided to men with PC at any point in care. This included nutrition education sessions, cooking classes, individual counselling and online or telephone services that are available to men with PC.

The predominant search strategy was to contact each of the ten provincial cancer agencies that provide cancer services for Canadians. We asked personnel at the cancer agencies to share information on other relevant nutrition services outside of their organization. Contact with identified organizations was attempted through email or telephone up to three times. This was supplemented by consulting a BC Cancer resource librarian for knowledge on existing nutrition services; and an online search using the following keywords “prostate cancer” and “nutrition” or “diet” and “services” using the Google search engine and limited results to those in English and Canadian organizations. The search was inclusive of services up to August 2019. A data collection form was used to collect information on the scope of the program, the target audience and the mode of delivery of nutrition services (available in the Additional file 1. Environmental Scan Data Collection Form).

### Scoping literature review

We conducted a scoping literature review to describe best practices and research related to delivering nutritional services to men with PC including men with a history of PC and current diagnosis. The PRISMA checklist was used to define the population and guide the search and data collection strategies. A reference librarian at the University of British Columbia guided the database selection and search strategy, which relied on Embase, Medline and CINAHL electronic databases to identify relevant articles. MeSH terms and keywords included variations of the terms “prostate cancer” and “nutrition services” as detailed in the Additional file 1. We reviewed the references of articles to identify additional articles not captured by the initial search. We also searched Clinicaltrials.gov to identify ongoing and planned studies that included nutritional services.

Articles were included in the review if they met the following inclusion criteria 1) participants were men with a PC diagnosis; either exclusively or a proportion of the overall study population; 2) studied a nutrition service or an education program; 3) the service or program was provided after PC diagnosis; 4) the article was in English and 5) articles published within the past 10 years (between 2007 and 2018). Articles were excluded if there was insufficient information on the nutrition services or program that prohibited extraction of key information.

We used Mendeley referencing software version 1.17.9 to upload search results and remove duplicate articles. The primary reviewer conducted a title and abstract review of each article followed by a full text review to select articles for inclusion. A secondary reviewer repeated this process on a random 25% subset of articles. The agreement between the first and second reviewer was 97% for the title and abstract screening of articles from the initial search. The agreement was 57% for the full text screening of articles included from the title and abstract screening. Due to the discrepancy, the reviewers met in-person to re-review the screening process, discuss articles in disagreement and identify the source of the disagreements. The full text review was subsequently repeated and agreement was reached for all articles.

From each article, we abstracted summaries of key points, the type of service provided (diet and exercise; nutrition; diet and mindfulness; clinical program) or delivery of nutrition services (home-based, group education, individual counselling, other), findings and study limitations and strengths. The data abstraction form is available in the Additional file 1.

### Analysis

Descriptive statistics are reported as means for continuous variables and as number and percentage for categorical variables for all four aims; patient evaluation, the health professional survey, the environmental scan and scoping literature review of nutrition services for men with PC.

Comparisons of categorical variables were performed using Chi squared tests while comparisons between continuous variables were performed with t-tests. Fisher’s exact tests were performed when the assumptions for Chi squared tests were not met. Significance was determined at *p* < 0.05. Analyses were performed with R version 3.4.3 (R Foundation for Statistical Computing, Vienna, Austria). Analyses specific to each study approach are described below.

### Evaluation of nutrition education sessions

Questions with missing data (non-response) were excluded from the analysis (*N* = 3, 13, 45, 27, and 6 participants for questions one through five, respectively). Responses were summarized for all respondents as well as separately for patients and partners. Differences in responses between patients and partners and between patients who attended sessions with a partner versus those who attended alone were compared using chi square tests or Fisher’s exact tests for yes/no questions and two sample t-tests for Likert scale questions. Open-ended questions were analyzed using deductive and realist qualitative methods [32]. Deductive analysis uses qualitative data to identify themes specifically related to the research question and a realist approach describes the experience and reality of participants as reported in the data [32]. Responses were coded as key words and organized by emerging themes that were decided and summarized based on the frequency of occurrence. Themes and the total number of responses for each theme are reported and used to provide more detail and richness to the data.

### BC Health professional survey

Question three, which asked about perceived demand for nutritional support for PC patients had 1 non-response which was excluded from the analysis, all other questions were complete. Responses for each question were summarized as proportions and compared across professions (physicians, radiation oncologists, medical oncologists, registered dietitians and researchers). Responses between dietitians and physicians, urologists, radiation oncologists and medical oncologists together were compared with chi squared tests and fisher’s exact test. Each question had the option to provide an open-ended response (‘other, specify’). These responses were thematically analyzed in the same manner as the nutrition education session.

### Environmental scan and scoping review

Information was categorized based on common themes including type of service/intervention, target audience and for the characterization of existing services; geographic region.

## Results

### Nutrition session evaluation

Between November 2013 and September 2016, 14 nutrition education sessions were delivered and 207 completed evaluation forms from 135 men with PC and 72 partners were collected. Evaluation responses are shown in Table 1. All participants reported that the session was easy to understand. The majority (88%) reported that the session was an appropriate length and 87% did not feel there was any information missing from the session. When asked about the inclusion of their partners in the education session, 94% of participants (patients, partners and patients who did not attend with a partner) agreed that inclusion was useful. Overall, 63% of participants found the session *very beneficial* with a mean rating of 3.6 (SD = 0.52) out of 4 for both patients and partners. Responses between patients and partners and between patients who attended with their partner and those who did not were similar for all questions with the exception of question four which asked if the inclusion of partners was valuable. The vast majority of patients (92%), partners (99%) and patients with partners (98.5%) responded ‘yes’ and thus cell counts for ‘no’ were too few for comparison.

**Table 1:** Patient and partner responses to the nutrition session evaluation

Participant	Response		*p*-value
Q1. Was the material presented in a clear fashion and easy to understand?			
	Yes, N (%)	No, N (%)	
Patient	133 (100)	0	
Partner	71 (100)	0	–
Patient with partner	71 (100)	0	
Patient without partner	39 (100)	0	–
Q2: Is there information you feel that was missed and should be included?			
	Yes, N (%)	No, N (%)	
Patient	18 (14)	113 (86)	
Partner	7 (11)	56 (89)	0.61
Patient with partner	8 (11.3)	63 (88.7)	
Patient without partner	8 (21.6)	29 (78.4)	0.15
Q3: Would you prefer the session be longer or shorter?			
	Yes, N (%)	No N (%)	
Patient	14 (13)	94 (87)	
Partner	6 (11)	48 (89)	0.32
Patient with partner	13 (12.4)	92 (87.6)	
Patient without partner	5 (14.7)	29 (85.3)	–
Q4: Did/would you find the inclusion of partners and/or family members valuable?			
	Yes, N (%)	No, N (%)	
Patient	103 (92)	9 (8)	
Partner	67 (99)	1 (1)	–
Patient with partner	128 (98.5)	2 (1.5)	
Patient without partner	21 (72.4)	8 (27.6)	–
	Mean rating out of 4 (SD)		*p*-value
Q5: Overall, how beneficial did you find the session?			
Patient	3.62 (0.54)		
Partner	3.64 (0.49)		0.86
Patient with partner	3.70 (0.55)		
Patient without partner	3.53 (0.50)		0.10

Comparisons between patients and partners and patient with partner and patient without partner for questions 1–4 with chi-square and question 5 with two-tailed t-test. Missing p-value indicates inadequate sample size for analysis

Thematic analysis of the qualitative data from the evaluation forms indicated a high level of satisfaction with the session. Other themes that emerged from the comments suggested that inclusion of partners was useful for processing the information presented during the session (*n* = 16, 30% of comments), helpful for implementing and supporting dietary changes at home (*n* = 34, 63% of respondents) or both (*n* = 5, 9%). Comments identified perceived information gaps in the nutrition education session among 27% of respondents such as the need for more information on the role of specific dietary components (e.g. sugar, genetically modified food, animal protein, and supplements), as well as individual concerns among 12% of respondents (e.g. food sensitivities, diet for specific PC treatments), and practical meal planning tips and suggestions. Several participants (12%) also indicated that they would like access to further nutrition services, and 5% further specified they wanted access to one-on-one dietary counselling.

### BC health professionals survey

The survey was sent to 56 health professionals in BC including 41 physicians (urologists (*n* = 24), radiation oncologists (*n* = 12), medical oncologists (*n* = 5)), registered dietitians (*n* = 13) and researchers (*n* = 2). Thirty-eight healthcare professionals responded to the survey for a response rate of 68%. The questions and response rates by each profession are shown in the Additional file 1: Table S1. BC Health Professional survey response rate across professions.

The majority of health care professionals that responded (85%) reported that men with PC have expressed nutrition related concerns to them. Over 60% of health professionals agreed that men with PC are in need of more nutritional support, 16% reported that current nutrition services are sufficient and 24% responded with “other”. Of those who responded with “other”, nearly half stated they were unaware of current nutritional services for men with PC. One reported that men require specific food and nutrition skills for PC. The remaining believed there was not a high demand for nutrition information among men, that there were sufficient services but men were unaware of current programs or that nutrition support was not as critical for men with PC compared with other cancers. There were no significant difference between responses from physicians and dietitians when asked about men with PC’s interest and need for nutrition services.

Most health professionals responded that nutrition services should focus on weight management (61%) and reducing the risk of PC progression (53%). However, there was inter-professional disagreement with respect to the focus of nutrition services - 90% of registered dietitians believed services should focus on reducing the risk of PC progression, whereas 75% of physicians believed the focus should be on weight management. The majority (67%) responded that nutritional services should be available to men multiple times after diagnosis through consecutive group education sessions (66%), online resources (66%) or individual nutritional counselling with a registered dietitian (50%). The overall themes that emerged from the open-ended questions suggested that 1) nutrition services should be available in different forms to facilitate individual needs (*n* = 12, 34%) and 2) there is a need for more nutrition services and organizational capacity to deliver services (*n* = 8, 23%). Health professionals specified a lack of funding (*n* = 2, 6%) and dietitian resources (*n* = 1, 3%) as barriers to implementing nutrition services for men with prostate cancer. A summary of the thematic analysis is available in the Additional file 1: Table S3. Summary of themes from the health professional survey.

### Environmental scan of existing nutritional services for men with PC

A total of 30 organizations that provide nutritional services across Canada were identified. Of the organizations identified, 22 provided information on their services. Six of the 22 (27%) organizations offered nutritional services specifically targeted to men with PC. Of these, four offered group education sessions and three offered online services (nutrition videos and online portal with nutrition information). The PC-specific group education sessions were offered through organizations in BC (*n* = 2), Alberta (*n* = 1) and Nova Scotia (*n* = 1). The content focused on diet modifications for different PC treatments, review of diet and PC evidence and promotion of a healthy diet. Six nutritional guides for men with PC were identified through the online search, three from national agencies, two from BC Cancer and one from a cancer centre in Ontario. The remaining 16/22 organizations offered nutrition services for cancer patients including but not specific to men with PC. A variety of services such as cooking classes, group nutrition education sessions, online resources, dietitian telephone services, wellness retreat and individual counselling were available through these organizations.

### Scoping review

The literature search identified 1030 articles and interventions through databases (*n* = 999), references lists (*n* = 25) and www.clinicaltrials.gov (*n* = 6). After the title and abstract review, 33 articles and interventions were included in the final scoping review. Of these articles, 27 were primary studies while the remaining six articles were secondary analyses of the primary studies (Table 2). An exception to the inclusion criteria of publication in the last 10 years was made for four studies, two of which provided details on study design and methodology needed to assess studies captured in the search. The other two were included as they represented a significant contribution to the body of evidence that was not captured in more contemporary studies.

**Table 2:** Studies identified in the nutrition services scoping review

Author	Mode of nutrition services	Focus of nutrition services	Design	Aim	Key Findings
Demark-Wahnefried [16, 33]	Home-based	Diet and exercise	RCT	To determine if custom exercise and nutrition print material is effective in promoting lifestyle changes for people living with breast or prostate cancer.	The FRESH START intervention arm demonstrated significant improvement in practicing goals, exercise, dietary intake and BMI compared to the control.
Christy [17]	Home-based/ Individual counselling	Diet and exercise	RCT	To assess the efficacy of the FRESH START trial 2 years post baseline.	The intervention arm sustained significant improvements in dietary intake and diet quality compared to the control.
Mosher [33, 34]	Mail	Diet and exercise	RCT	To examine changes in self-efficacy as a mediator of the FRESH START intervention.	Changes in self-efficacy significantly mediated the effect of the intervention on dietary outcomes.
Morey [18] & Snyder [35]	Home-based	Diet and exercise	RCT	To determine if home-based multi-behaviour interventions can improve functional decline in older cancer survivors.	The intervention arm had a significant reduction in the rate of functional decline and improvements in physical activity, dietary behaviours and overall quality of life.
Demark-Wahnefried [36, 37]	Home-based	Diet and exercise	RCT	To determine if a home-based diet and exercise intervention can improve physical functioning in older adults with breast and prostate cancer.	At 6 months the intervention group improved diet quality and self-efficacy to exercise but these changes were not maintained post intervention.
Lebret [38]	Home-based	Diet and exercise	Longitudinal survey	To assess the use of a diet and exercise tool-kit in improving well-being for men with PC on ADT.	The majority of patients and urologists reported satisfaction with the tool-kit and the majority of patients reported implementing guidelines from the toolkit.
O’Neil [19]	Home-based	Diet and exercise	RCT	To assess if a home-based diet and exercise intervention can help minimize side effects of ADT for men with PC.	At 6 months the intervention arm demonstrated significant improvements in body composition, dietary intake, functional capacity but not fatigue, stress or QoL.
Bourke [39]	Group education	Diet and exercise	RCT	To assess the effect of lifestyle interventions on QoL, diastolic blood pressure and fatigue in men with PC on ADT.	At completion the intervention arm had significant improvements in QoL, fatigue and exercise tolerance however only improvements in fatigue, exercise tolerance were sustained at 6 months.
Bourke [40]	Group education	Diet and exercise	RCT	To assess the feasibility of a tapered supervised exercise and diet intervention for men with PC on ADT.	The intervention arm showed significant improvements in dietary intake, exercise, and fatigue however there were high attrition rates at 6 months.
Bourke [41]	Group education	Diet and exercise	RCT	To qualitatively evaluate tapered exercise and diet intervention for men with PC on ADT.	Participants reported benefiting from the intervention both physically and psychologically. Participants reported benefits from dietary education but found adherence to guidelines difficult.
Carmody [42]	Group education	Diet	RCT	To assess if dietary interventions can improve dietary intake, QoL and PSA velocity in men with PC.	The intervention arm demonstrated improvements in diet quality/ intake and QoL. No significant changes were observed for PSA velocity.
Nguyen [43]	Group education/ Individual counselling	Diet and mindfulness	Pre-post	To measure changes in dietary intake and PSA velocity following a dietary and stress reduction intervention in men with recurrent PC.	Following the intervention the participants significantly improved dietary intake however no significant reductions in PSA rise were observed.
Davison [44]	Group education	Diet	Pre-post	To measure the impact of a nutrition intervention on calcium and vitamin D intake for men with PC on ADT.	Following the intervention there were no significant increases in calcium and vitamin D through dietary intake however there were significant increases in through supplement intake.
Hebert [45]	Group education/ Individual counselling	Diet and exercise /Diet and mindfulness	RCT	To assess the effects of lifestyle interventions on PSA levels and reducing the risk of PC recurrence.	The intervention arm did not show any significant changes in PSA levels however they did demonstrate significant improvements in dietary intake.
Ferguson [46]	Group education	Comprehensive program	Implementation study	To evaluate the implementation and early impact of a nurse-led survivorship program for men with PC.	The program had high participation rates with 90% attendance. Feedback from participants suggests high user satisfaction and reported QoL improvement.
Baguley [47]	Individual counselling	Diet	RCT	To assess the efficacy of a Mediterranean-style nutrition intervention on cancer related fatigue and QoL in men with PC on ADT.	Improvements in QoL and fatigue were not significant, however the intervention arm did demonstrate significant changes in weight.
Baguley [48]	Individual counselling	Diet and exercise	RCT	To assess the efficacy of high intensity interval training in addition nutrition therapy on cancer related fatigue in men with PC on ADT.	N/A
Aggarwal [49]	Individual counselling	Comprehensive program	Pre-post	To assess the adherence to a multidisciplinary clinic for men with PC on ADT and if the intervention can lessen the metabolic impacts of ADT.	Participation and adherence to the clinic was high with 95% adherence. The metabolic impact of ADT was minimal during the intervention.
Joly [50]	Individual counselling	Diet and exercise	Pre-post	To assess the impact of a nutrition and exercise intervention on body composition and physical function in frail men with PC on ADT.	After 3 months of ADT participants had improved timed-up-and-go test while other measures of physical function and body composition remained stable.
Focht [51]	Individual counselling	Diet and exercise	RCT	To assess the feasibility and preliminary efficacy of implementing a group-mediated cognitive behavioural lifestyle intervention for men with PC undergoing ADT.	N/A
Chan [46]	Other	Diet and exercise	Program summary	The purpose of TrueNTH is to create an international partnership and develop interventions to improve the physical and mental well being of PC survivors.	Canada, the U.S.A, Australia and the U.K. are developing lifestyle interventions and programs for men with PC and will evaluate implementation approaches.
Cosby [52]	Group education	Diet	Pre-post	To evaluate the effectiveness and satisfaction of a weekly diet and PC group education session on meeting information needs in promoting healthy body weights.	Significantly improved nutrition knowledge post session. Participants reported high satisfaction rates, usefulness of information, the importance of information and the value of group learning.
Golubić [53]	Group education	Comprehensive program	Pre-post	To evaluate the effectiveness of Lifestyle 180, a comprehensive, lifestyle program, on chronic disease risk factors and QOL in cancer survivors.	Significant improvements in weight loss and WC, BMI and measured biomarker. Reported reduction in lipid lowering and BP medication. Reported improvements in perceived stress and QOL.
Takada [54]	Group education	Diet	NRCT	To evaluated the efficacy of nutrition education on changes in daily rectal volume and dose during IMRT treatment.	Patients who attended the nutrition education session had decreased variations in rectal volume and treatment dose compared to controls resulting in more reproducible results.
Ongoing clinical trials:					
Goulart [55]	Group education	Diet and exercise	RCT	To evaluate the multidisciplinary Living Well on Androgen Deprivation Therapy program on QoL, physical factors and biomarkers of secondary diseases in men with PC on ADT.	N/A
Kellogg Parsons [56]	Individual counselling	Diet	RCT	To assess the impact of a telephone based dietary intervention on disease progression in men with PC on active surveillance.	N/A
McArdle [57]	Individual counselling	Diet and exercise	RCT	To assess the feasibility of a nutrition and exercise counselling intervention on reducing the incidence of metabolic syndrome in men with PC on ADT.	N/A
Maliski [58]	Individual counselling	Diet and exercise	RCT	To assess if counselling on lifestyle changes in addition to standard medical care can prevent heart problems and diabetes in men with PC on ADT.	N/A
Lee [59]	Home-based	Comprehensive program	RCT	To assess if the efficacy of a web and mobile based lifestyle intervention on physical function, strength, body composition and QoL.	N/A
Aggarwal [49]	Individual counselling	Comprehensive program	RCT	To assess if individualized counselling helps improve patient understanding, satisfaction, quality of life and anthropometrics.	

Abbreviations: *ADT* Androgen deprivation therapy, *NRCT* Non-randomized controlled trial, *QOL* Quality of life, *RCT* Randomized, controlled trial, *PC* Prostate cancer, *WC* Waist circumference, *BP* Blood pressure, *IMRT* Intensity-modulated radiotherapy. Grey shading indicates secondary analysis of a study that was also captured in the scoping review and were therefore not considered when calculating the type and focus of interventions

The majority of studies (63%) used a randomized controlled study design [16, 18, 19, 33, 35, 36, 39, 40, 42, 45, 47–49, 51, 55–59] while the remaining studies (22%) used pre-post [43, 44, 49, 50, 52, 53], longitudinal (4%) [38], implementation (4%) [46], a program summary design (4%) [60] and non-randomized control trial (4%) [54]. The length of study interventions ranged from a single time point to 24 months. Men with PC who were treated with ADT or were to receive ADT were the most common population (55%) [19, 38, 39, 43, 44, 47–51, 55, 57] followed by men post PC diagnosis (30%) [42, 43, 45, 46, 56, 60], and different cancer types including men with PC (15%) [33, 35, 36]. The majority (52%) of interventions were combined diet and exercise programs [19, 33, 35, 36, 38–40, 43, 48, 50, 51, 55, 57, 60]. Six (22%) [42, 44, 47, 48] exclusively provided nutritional services, while the remaining (18%) offered nutritional services as part of a comprehensive clinical program [46, 49], diet and mindfulness services (4%) [43], or diet, mindfulness and exercise services (4%) [45]. The format in which services were delivered included individual nutritional counselling (33%) [43, 47–51, 56], group education sessions (33%) [39, 40, 42, 44, 46, 55], both individual and group sessions (7%) [43, 45], home-based services (22%) such as mailed educational material [19, 35, 36, 38, 40], and multiple forms of delivery (4%) including classes, home-based and individual counselling [60]. Additional details of the studies identified can be found in Table 3.

**Table 3:** Summary of studies identified in scoping review

Study characteristics	Number (N)	Proportion (%)
Design		
RCT	17	63
Pre-post	6	22
Other	4	15
Mode of nutrition service		
Group education	9	33
Individual counselling	9	33
Home-based	6	22
Group / individual counselling	2	7
Other	1	4
Focus of intervention		
Diet and exercise	14	52
Diet	6	22
Comprehensive program	5	18
Diet, mindfulness and exercise	1	4
Diet and mindfulness	1	4
Population		
Men with PC treated with ADT	15	55
Men with PC	8	30
Cancer patients	4	15

Of the 27 studies identified in the review, 18 were complete and had published their findings (Table 2). The remaining nine studies were in progress at the time of the literature review. Of the completed studies, 78% reported improvements in one or more of the following measures: dietary intake/ diet quality, body composition, self-efficacy, quality of life, fatigue, practicing health behaviour goals and physical function/ exercise. Christry et al. was the only study that measured the long-term impact of nutrition services and found sustained improvements in diet quality, energy intake, and saturated fat intake 2 years following a 10-month home-based diet and exercise intervention [17]. All studies that examined qualitative outcomes (*n* = 4) such as feasibility, adherence and participant satisfaction, reported positive findings [38, 40, 46, 49]. Common facilitators of dietary behavior change and/or patient satisfaction were group education [1, 42] and peer support [46, 59]. Other studies emphasized the importance of individualized nutrition services [9, 50, 52]. Common limitations included the use of self-reported measures, healthy participant bias, small sample size, short follow up period and lack of generalizability to the overall PC population. Our interpretation was limited by studies of participants with various cancer types (e.g. breast, colorectal, prostate) that did not provide cancer-specific results. This limits our understanding of the effect of these interventions on men with PC.

## Discussion

To our knowledge this is the first report to provide a multifaceted approach to capture the need for nutritional services focused on men with PC. This approach is critical for informing and delivering evidence-based health services. Findings from multiple perspectives suggest that men with PC have an unmet need for nutritional information during supportive care. For instance, a need for additional nutrition services and support was identified by respondents to the BC health professional survey, few services for men with PC exist and even among those with access to nutrition education, there was indication of wanting more support. However, findings also indicate that survivorship clinics and cancer care centres vary widely in the range of nutritional services provided to men with PC, and few are specific to PC. Although generally, nutrition interventions were effective, nutrition interventions for men with PC published in the literature are heterogeneous with respect to design, mode of delivery and content, making it difficult to identify best practices. Standardized approaches would facilitate discovery and potentially implementation of effective PC care, however, the complexity of such an undertaking would be substantial.

Each of the four approaches taken in this study provided important findings that can inform supportive care programming. The evaluation forms from the nutrition education session demonstrated overall high satisfaction with the PCSC Program’s existing in-person group-based educational session. Together with the qualitative feedback expressing a desire for more nutrition related information, this demonstrates a patient-perceived need for nutrition services among men with PC. A previous study among recently treated men with PC also reported group education on general PC knowledge including nutrition was well accepted and resulted in increased knowledge [61]. Limitations to the group-based format included the need for more personalized and additional nutrition information. The need for more information is echoed in the literature including a study by Taylor et al. [62] that reported more than 50% of cancer patients would like more information on managing illness. However, providing additional nutrition supportive care for men with PC within a public healthcare setting is a challenge, especially given this requires a proactive versus reactive approach as patients generally are not malnourished. Findings from the other perspectives studied herein may provide direction moving forward.

The findings from the BC health professional survey aligns with a comprehensive review of educational needs of cancer patients by Rutten et al. [63] who reported that cancer-specific and treatment-related information (of which nutrition is relevant to both) was required continually from diagnosis, treatment and post-treatment. Of note, there were differences between professions with respect to the content of nutrition services. Dietitians indicated that information should focus on the role of diet to reduce the risk of PC progression while medical oncologists, radiation oncologists and urologists believed that diet for weight management was the most important topic. We speculate that the differences in opinion may reflect questions commonly posed to dietitians by patients which is supported by the qualitative feedback on the nutrition evaluation session that requested more information on different dietary components and prostate cancer and notably there was no mention of more information for weight maintenance purposes. The focus on weight management by oncologists and urologists may reflect the relevance of weight management in clinical practice since increased body weight is linked to an increased risk of future prostate cancer mortality in those who are cancer free and increased risk of biochemical relapse after primary therapy [64, 65]. In addition, increased weight gain is a known side effect of ADT that can be associated with metabolic syndrome and have downstream adverse effects on health [66]. Further work is needed to understand the differing perspectives in health professional disciplines to support the development of need-driven nutrition services.

Meeting the nutritional needs of men with PC in a busy healthcare setting is a challenge that is likely reflected by the small number of existing services specific to PC in organizations across Canada. Among the six we identified, four were in-person group education sessions. Group education has been shown to be an effective means of delivering health information in a comparable and potentially more efficient and cost-effective manner than individual education [67, 68]. However, in-person sessions may pose accessibility problems due to geographic and logistical barriers. The remaining two organizations provided nutrition services online, which has potential to provide supportive care to a larger number of men with PC and to overcome barriers to access. Although the efficacy of the online services was not assessed, literature suggests telemedicine education can be equally effective as in person education [69]. Between 2028 and 2032, the number of new cases of PC is projected to almost double in Canada [70]. The impact on healthcare services for the growing number of men living with PC will be substantial. Alternative modes of delivery of patient information will therefore be critical.

The scoping review identified three main methods of delivering nutrition services to men with PC; home-based services, individual counselling, and group education classes. This substantiated what healthcare professionals in our survey considered the best modes of delivery, suggesting supportive care programs should consider offering flexible formats for nutrition services when possible. Although this paper is focussed on nutrition, diet is just one part of a healthy lifestyle. The generally positive findings reported by studies in the review such as feasibility, adherence and user satisfaction were not specific to the dietary component. Thus, we suggest that supportive care programs offer comprehensive healthy lifestyle services including nutrition. It is also important to highlight the difficulty in identifying ‘best practice’ and comparative success of approaches from the literature to inform nutrition services as studies were heterogeneous in design, target population and in primary outcome. Unsurprisingly, existing nutrition services identified in this study generally did not reflect interventions reported in literature, nor the perspectives of health care professionals with respect to content and timing of services. This confirms the well documented gap between research and practice [71].

There are several limitations to our study. Patient perspectives on nutritional services were assessed from data already collected through the PCSC programs education session evaluation form that was not designed to assess perspectives on broader nutrition services. The form also did not collect demographic information as the purpose is quality improvement and not research. Therefore, participants who attended the nutrition education session may not be representative of the general PC population. Patient engagement through focus groups and surveys would provide a deeper understanding of nutritional needs. The survey of health professionals used purposive expert sampling to capture insight on nutrition services from those working directly in PC-based healthcare or research in BC. Questions were developed by the researchers and not validated. This approach was used as our aim was exploratory in nature and required a focus on individuals with expertise providing care for men with PC. However, it also introduces researcher bias and a non-representative sample as it was confined to BC. One of the strengths of our study was the use of a multi-faceted approach that considered patient and health care perspectives, existing services and the evidence base on nutrition and PC. Incorporation of each of these areas is key for sustainable, effective nutrition services, and supportive care services more broadly.

## Conclusion

As evident from the environmental scan, there are limited nutritional services targeted to the PC population despite the high prevalence of PC in Canada, and the effectiveness of nutritional services. It is perhaps unsurprising then that men with PC and PC-healthcare professionals identified a need for more nutrition services. Nutrition services should consider flexibility in delivery format, support at multiple times throughout survivorship, as well as embedding nutrition as part of overall supportive care. The provision of such support will be a challenge within a public healthcare system where nutrition services are generally prioritized for those who are malnourished, which is uncommon among men with PC. New models of care with supplemental funding may help to close the gap between the needs of men with PC and current standard of care.

## Supplementary information

**Additional file 1.** BC Health Professional Survey Questions. Environmental Scan Data Collection Form. Supplementary Table 1: BC Health Professional survey response rate across professions. Supplementary Table 2: Scoping Literature Review search strategy. Supplementary Table 3: Summary of themes from the health professional survey.

## Data Availability

Data generated or analyzed during this study are included within the article and its supplementary information files.

## Abbreviations

ADT: Androgen Deprivation Therapy
BC: British Columbia
BCCA: British Columbia Cancer Agency
PC: Prostate Cancer
PCSC: Prostate Cancer Supportive Care
VGH: Vancouver General Hospital
