# Supplementary material for: Assessing the nutritional needs of men with prostate cancer
Source: Nutr J. 2019 Dec 2;18:81. doi: 10.1186/s12937-019-0506-7 (PMC6889583; doi:10.1186/s12937-019-0506-7)
Supplement: Supplementary file 1 — Additional file 1. BC Health Professional Survey Questions. Environmental Scan Data Collection Form. Supplementary Table 1: BC Health Professional survey response rate across professions. Supplementary Table 2: Scoping Literature Review search strategy. Supplementary Table 3: Summary of themes from the health professional survey. [file 12937_2019_506_MOESM1_ESM.docx]

**Assessing the Nutritional Needs of Men with Prostate Cancer**

Kaitlin McLaughlin^1^, Lindsay Hedden^1,2,3^, Philip Pollock^3^, Celestia Higano^3,4,5^, Rachel A Murphy^1*^

^1^School of Population and Public Health, University of British Columbia, Vancouver, BC, Canada

^2^Centre for Clinical Epidemiology and Evaluation, University of British Columbia, Vancouver, BC, Canada,

^3^Vancouver Prostate Centre, Vancouver, BC, Canada

^4^Department of Urologic Sciences, University of British Columbia, Vancouver, BC, Canada

^5^University of Washington, Fred Hutchinson Cancer Research, Seattle, WA, United States

**BC Health Professional Survey Questions**

**Question 1. Are you aware of any nutrition programs for prostate cancer patient’s other than the PCSC program?**

Yes

No

If yes, specify ____________

**Question 2. Have prostate cancer patients expressed nutrition related concerns with you?**

Yes

No

N/A

If yes, specify ____________

**Question 3. What is your impression of the demand for nutritional support among prostate cancer patients?**

Need less support

Current support is sufficient

Need more support

Other, specify ________________

**Question 4. When should nutrition information and services be provided to prostate cancer patients?** *Select all that apply*

At diagnosis

Before treatment

After treatment

Never

Other, specify ________________

**Question 5. What type of content should be the focus of nutrition services provided to prostate cancer patients and their partners?** *Select all that apply*

Nutrition for prostate cancer treatment/therapy

Weight management

Prostate cancer progression

Chronic disease management

Other, specify _______________

**Question 6. What mode of delivery do you think is best to provide nutrition information to meet the needs of prostate cancer patients?**

Consecutive group education sessions

Online resources

Brochures

Individual consults with a Registered Dietitian

A combination

Other, specify _____________

**Supplementary Table 1. BC Health Professional Survey Response Rate Across Professions**

| **Role** | **Number sent** | **Number received** | **Response rate (%)** |
| --- | --- | --- | --- |
| Researchers | 2 | 2 | 100 |
| Registered dietitians | 13 | 10 | 77 |
| Urologists | 24 | 16 | 67 |
| Radiation oncologists | 12 | 6 | 50 |
| Medical oncologists | 5 | 2 | 40 |
| ^a^Unknown |  | 2 | 40 |
| **Total** | **56** | **38** | **68** |

^a^Respondent did not indicate their profession on the anonymous survey

**Environmental Scan Data Collection Form**

1. Organization
2. Location (Province)
3. Contact Information
4. Date and method of contact
5. Name of program or service offered
6. Does this service include nutrition education?
7. Is this service prostate cancer specific?
8. How is this service delivered? (i.e. individual counselling, online, etc.)
9. Is it part of another survivorship program?
10. What is the scope of the service?

**Supplementary Table 2. Scoping Literature Review search strategy**

| **Database:** | **Date searched** | **Search Strategy** |
| --- | --- | --- |
| **Embase (Ovid)** | Aug 3 2019 | 1. exp prostate cancer/ 2. prostate cancer/ or prostate cancer.mp 3. (nutrition service* or nutrition program* nutrition education* or survivorship program* or health promotion* or dietitian* or dietician*). Mo [mp=title, abstract, heading word, drug trade name, original title, device manufacturer, drug manufacturer, device trade name, keyword, floating subheading word] 4. exp dietary service/ 5. 1 or 2 6. 3 or 4 7. 5 and 6 |
| **Medline (Ovid)** | Aug 3 2019 | 1. exp Prostatic Neoplasms/ 2. prostate cancer.mp. 3. exp Dietary Services/ 4. (nutrition service* or nutrition program* nutrition education* or survivorship program* or health promotion* or dietitian* or dietician*).mp [mp.=title, abstract, original title, name of substance word, subject heading word, keyword heading word, protocol supplementary concept word, rare disease supplementary concept word, unique identifier, synonyms] 5. 3 or 4 6. 1 or 2 7. 5 and 6 |
| **CINAHL (EBSCOhost)** | Aug 3 2019 | S1. (MH“Prostatic Neoplasms+”)  S2. (MH“Nutrition Services+”)  S3. prostate cancer  S4. nutrition service* or nutrition program* or nutrition education* or survivorship program* or health promotion* or dietitian* or dietician*  S5. S1 OR S3  S6. S2 OR S4  S7. S5 AND S6 |
| **Clinical trials.gov** | Aug 3 2019 | 1. prostate cancer 2. nutrition services 3. 1 and 2 |

**Scoping review data extraction form**

| Data extraction form: |
| --- |
| 1. Author and reference details    1. (contact info, year, journal, volume, DOI etc.). 2. Type of study design    1. RCT, longitudinal cohort, case control, etc.    2. How many people were included in the study 3. Objectives    1. Primary objective of the study/ report 4. Participant characteristic    1. Demographics (age, ethnicity)    2. Location    3. If partners were included or not    4. Were any other participants included in the study that isn’t our targeted population 5. Intervention/ service provided    1. Context of intervention (what is provided ie: advice, care plans etc.)    2. Is this service part of another program    3. Mode of delivery    4. Who is providing the service    5. Is it individualized or standardized    6. Location of intervention    7. Cultural context 6. Outcomes    1. Primary: changes in quality of life and satisfaction of patient care, improved health outcomes.    2. Secondary: comorbidities, weight, feasibility of a program 7. Context    1. Type of health care system? 8. Methodological assessment/ limitations    1. Any limitations in the study design and analysis |

**Scoping Review Flow Diagram**


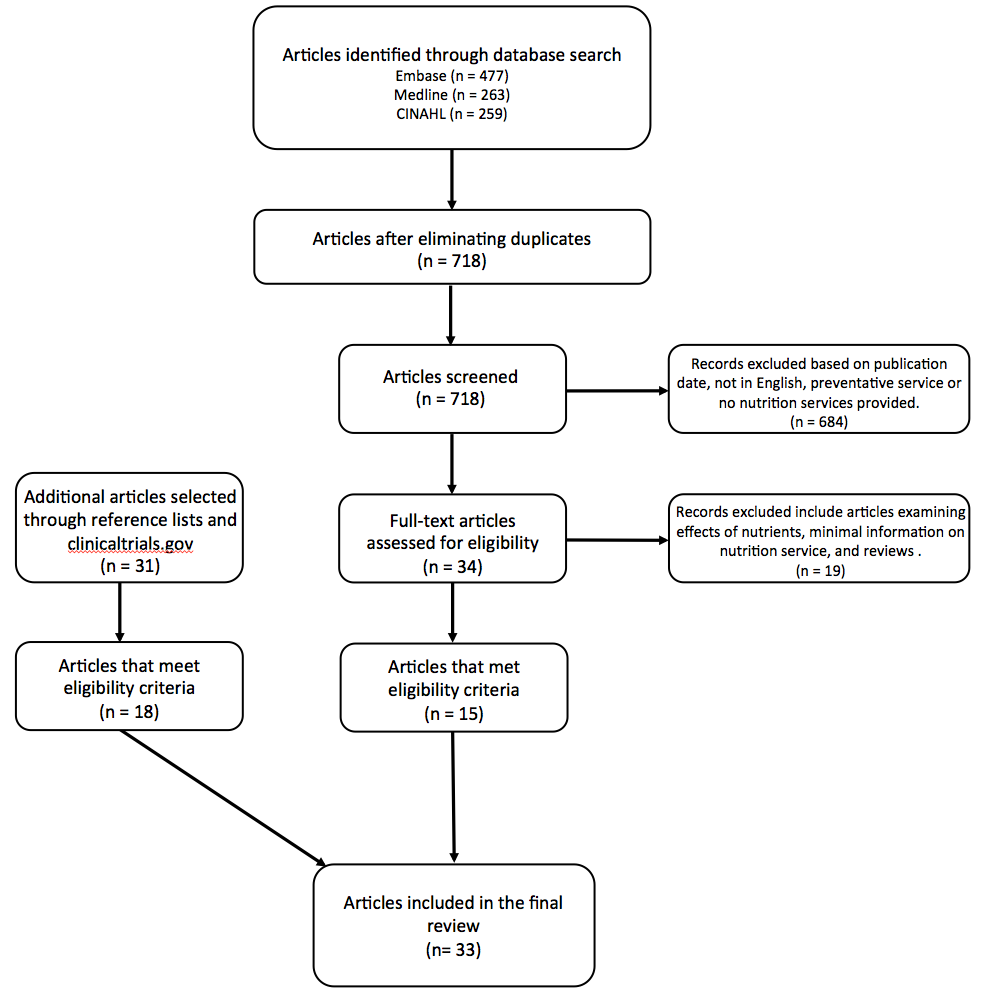


**PCSC Program- Nutrition for Prostate Cancer Patients Education Session Evaluation Form**

Date of Session Attended (Month/Day/Year): ________/________/________

I am a………..**prostate cancer patient** ☐ or **partner/family member of a patient** ☐

If you have a partner, did they participate in this session with you? No ☐ Yes ☐ N/A ☐

Was the material presented in a clear fashion and easy to understand?

No ☐ Yes ☐, If No, please explain:

______________________________________________________________________________________________________________________________________________________________________

Is there information you feel that was missed and should be included?

No ☐ Yes ☐, If Yes, please explain:

____________________________________________________________________________________________________________________________________________________________________

Would you prefer the session be longer or shorter?

No ☐ Yes ☐, If Yes, please explain:

______________________________________________________________________________________________________________________________________________________________________

Did/would you find the inclusion of partners and/or family members valuable?

No ☐ Yes ☐, Please explain:

______________________________________________________________________________________________________________________________________________________________________

Overall, how beneficial did you find the session? *(Circle one number)*

**0** ---------------------- **1** ---------------------- **2** ---------------------- **3** ---------------------- **4**

Not at all Somewhat Very beneficial

If there is anything else you would like to add, or other suggestions you would like to make about this session, feel free to add those comments on the reverse of this shee

Further comments:

**Supplementary Table 3**: Summary of themes from the health professional survey.

| **Main themes** | **Example of responses** |
| --- | --- |
| 1. Nutrition services should be available in different forms to facilitate individual needs | “A range of [nutrition] services should be available to create accessible care that accounts for individual preferred learning styles.” |
|  | “…There is a need to develop and implement culturally relevant nutrition education for prostrate cancer patients... Cultural adaptation to any aspect of service delivery for prostrate cancer patients would be very helpful.” |
|  | “Individualize content and modes of delivery to reach a broad audience; solicit views from diverse patients.” |
|  | “Different strokes for different folks.” |
| 2. Need for more nutrition services and organizational capacity to deliver services | “There is a demand for nutrition information that is not being met for this population. We are merely scratching the surface at meeting patient needs across their journey from diagnosis to survivorship…” |
|  | “I feel less informed because I only see [patients] who are in very acute risk, but I know there is a lot of questions from [patients] and would be nice to have more resources for them.” |
|  | “There appears to be a lack of awareness of the existing nutrition services…lack of resources for dietitians to provide individualized nutrition counseling during treatment and lack of support programs re: nutrition and lifestyle for patients and caregivers post treatment.” |
|  | “…I don't perceive that we have a good capacity, within [BC Cancer Agency], to meet a lot of the educational needs/wishes of patients…” |
|  | “Lack of funding is limiting the invaluable services available.” |

**Supplementary Table 4**. Summary of themes and sub-themes from the nutrition education session evaluation.

| **Main themes and sub-themes** | **Example of responses** |
| --- | --- |
| Satisfaction with education session | “This was an excellent seminar and very informative -I would highly recommend it to patients and partners.” |
|  | “Excellent presentation, useful and necessary.” |
| Inclusion of partners useful for: |  |
| Processing information | “Lots of info, it's good to have another person to intake some.” |
|  | “So we can pool what we learned (and trust that the other person wasn't fudging the facts).” |
| Supporting dietary change | “She is my home nutritionist and helps me with my discipline.” |
|  | “Really important so much easier when you and your partner understand the commitment to doing well.” |
| Requested for information on: |  |
| Specific dietary components | “Zinc? Herbal supplements” |
|  | “Didn’t mention connection of sugar to insulin and thus cancer.” |
| Individual concerns | “Is there any difference with a diet as applied to a prostatectomy?” |
|  | “Only thing to add would be food allergies/sensitivities” |
| Practical tips for implementation | “Actual menu suggestions would be very helpful.” |
|  | “More detail or follow up detail for actual implementation…” |
| Access to more nutrition services | “We need much more of this service, additional resources are required.” |
|  | “Very informative, more awareness of these programs, updates or other seminars.” |
| Individual counseling | “Would like one-on-one appointments. Thanks.” |
|  | “Would love to have additional one on one session where review food patterns (i.e. record for 2 weeks and then 30 min session with dietician).” |
